# Research on the chemical oxygen demand spectral inversion model in water based on IPLS-GAN-SVM hybrid algorithm

**DOI:** 10.1371/journal.pone.0301902

**Published:** 2024-04-11

**Authors:** Qirong Lu, Jian Zou, Yingya Ye, Zexin Wang

**Affiliations:** 1 College of Information Science and Engineering, Guilin University of Technology, Guilin, China; 2 Guangxi Key Laboratory of Embedded Technology and Intelligent System, Guilin University of Technology, Guilin, China; State University of New York at Oswego, UNITED STATES

## Abstract

Spectral collinearity and limited spectral datasets are the problems influencing Chemical Oxygen Demand (COD) modeling. To address the first problem and obtain optimal modeling range, the spectra are preprocessed using six methods including Standard Normal Variate, Savitzky-Golay Smoothing Filtering (SG) etc. Subsequently, the 190–350 nm spectral range is divided into 10 subintervals, and Interval Partial Least Squares (IPLS) is used to perform PLS modeling on each interval. The results indicate that it is best modeled in the 7th range (238~253 nm). The values of Mean Square Error (MSE), Mean Absolute Error (MAE) and R2score of the model without pretreatment are 1.6489, 1.0661, and 0.9942. After pretreatment, the SG is better than others, with MSE and MAE decreasing to 1.4727, 1.0318 and R2score improving to 0.9944. Using the optimal model, the predicted COD for three samples are 10.87 mg/L, 14.88 mg/L, and 19.29 mg/L. To address the problem of the small dataset, using Generative Adversarial Networks for data augmentation, three datasets are obtained for Support Vector Machine (SVM) modeling. The results indicate that, compared to the original dataset, the SVM’s MSE and MAE have decreased, while its accuracy has improved by 2.88%, 11.53%, and 11.53%, and the R2score has improved by 18.07%, 17.40%, and 18.74%.

## Introduction

Chemical Oxygen Demand (COD) is one of the indicators used to represent the degree of water pollution, which reflects the amount of oxidant consumed in the process of oxidizing a water sample. Currently, there are various methods for measuring COD in water, and among them, the model based on UV-visible spectroscopy has the characteristics of easy operation and data analysis. UV-visible-near infrared spectrophotometers and infrared spectrometers [1–6] are typically used for the qualitative analysis. Spectroscopic techniques [7–11] are also used for online monitoring and for studying the correlation between water indicators and spectral intensity. However, the spectral data are easily affected by various factors such as instrument response, sample preparation, and environmental noise, resulting in noise and biases. Pretreatment methods are essential to reduce noise and improve the correlation between spectral data and chemical composition. Methods such as Standard Normal Variate, Multiple Scattering Correction, Smoothing Filtering, Moving Average Filtering, First-Order Differentiation, Second-Order Differentiation, Wavelet Transformation, Standardization, and Normalization are widely adopted. In spectral modeling, Partial Least Squares (PLS) algorithm is typically combined with other algorithms to achieve better modeling performance [12]. For example, Kernel Partial Least Squares and Boosting PLS have been utilized to predict leaf water content [13]. In another study, PLS and Support Vector Machine (SVM) algorithms were used to detect trace element content in poultry manure [14]. Due to the collinearity of spectra, selecting the optimal modeling wavelengths is crucial. To this end, Ying Li [15] integrated swarm intelligence algorithms and the PLS algorithm to establish a model for detecting apple juice adulteration. Similarly, Cheng et al. [16] combined the genetic algorithm with the PLS model to obtain optimal modeling wavelengths.

SVM algorithms are typically used for chemical concentration detection [17–20]. C. Robert [21] used both linear and non-linear SVM models to identify complete beef and lamb meats. Similarly, H. Sun [22] combines the Kernel Principal Component Analysis and SVM to improve accuracy. However, compared to PLS, SVM requires larger datasets to achieve better training results. Furthermore, the high cost and large size of multi-functional spectrometers [23–25] make them impractical to acquire data in research groups with limited funding. Therefore, Generative Adversarial Networks (GAN) can be used for data augmentation [26–32]. Cao Z et al. [33] combined GAN networks in spectral data analysis to enhance analysis accuracy and mitigate overfitting. In response to the scarcity of rice seed spectral data, Qi et al. [34] generated rice seed spectral data to address the issue of limited samples. Based on this, a neural network model was established using three modeling methods: real data modeling, fake data modeling, and mixed modeling of real and fake data. Zhang M et al. [35] proposed a new data augmentation strategy based on the original GAN network to tackle the challenges of small sample sizes and imbalanced samples in hyperspectral image processing. J. Wang [36] utilized a trained CGAN model for data augmentation, resulting in a five-fold increase in the dataset. Additionally, Cai et al [37] utilized the spectrogram of samples as inputs and applied data augmentation based on GAN to generate additional training data. Miao et al. [38] utilized a GAN to generate highly similar and diverse synthetic samples for fault diagnosis.

After a comprehensive analysis, the experiment combines UV-Visible spectroscopy, Interval Partial Least Squares (IPLS) method, SVM, and GAN for COD concentrations analysis. First, the UV-Visible spectrophotometer is used to obtain the spectral intensity of COD samples. At the same time, six methods are used to preprocess the water data. Secondly, spectral data training and test sets are created, and IPLS is used to select the spectral range for modeling, which the entire spectral range is divided into 10 segments, and a PLS model is established for each range. Thirdly, the model with the highest accuracy will be selected. Subsequently, GAN is utilized to process both the original and preprocessed spectral data, generating additional data for modeling. Lastly, SVM models are constructed for both the original and generated spectral data to validate the feasibility of the GAN through modeling effects.

## Materials and methods

The study does not involve activities that require specific permits, such as working with endangered species or in protected areas. In accordance with local regulations and guidelines, no permits are required for this study.

### Instruments and reagents

The experiment uses a spectrum acquisition system composed of a hyperspectral imager, a quartz cuvette, and a tungsten lamp lighting source. The Ultraviolet (UV) spectrum of the water sample is obtained through this system produced by Beijing Puxi General Instrument Co., Ltd., and it has a wide wavelength range of 190nm~900nm and a high precision of ±0.3nm wavelength indication error. The detailed parameters are shown in the Table 1 below:

**Table 1:** Instrument performance indicators.

Indicators	Value of indicator
Model	TU-1950
Wavelength range	190nm~900nm
Wavelength error	±0.3nm
Repeatability of wavelength	Segment A (190nm~340nm): ≤0.10 nm; Segment B (340nm~900nm): ≤0.15nm
Spectral bandwidth	±20% (0.1nm~5nm continuously adjustable)
Transmittance ratio error	±0.3%
Drift	≤0.1%/h
Level	I

This instrument has functions such as photometric measurement, spectral scanning, quantitative determination, time scanning, spectral bandwidth scanning, DNA protein determination, and graphic processing. During the experiment, to ensure the accuracy of measurement results, a dark current calibration is used to eliminate some instrument noise. When measuring the absorbance or transmittance, baseline calibration is required. In this paper, the first step is to use the spectral scanning function to obtain the data of the COD samples, and then use Python for visual analysis to obtain the relationship between the solution concentration and the spectrum.

The sample pretreatment is as follows. The method is to take 0.8502g of potassium hydrogen phthalate solute, add distilled water to 1L, and stir until the solute is completely dissolved to obtain 1g/L COD solution. Based on this method, COD standard solutions with a concentration of 10-100mg/L are prepared in sequence. The specific implementation process of the paper is shown below.

### Collection and treatment of the water samples

The collection of surface water samples is an important step in environmental sciences, which is used to monitor water quality and to comprehend the state of pollution in water bodies. The following are the basic steps in surface water sampling:

1. **Determine the sampling points:** firstly, the location of the sampling points requires to be determined, which can represent the water quality in the Li River area.
2. **Prepare sampling equipment:** Bring appropriate sampling equipment such as sampling bottles and samplers. The equipment should be clean to avoid the contamination of the samples.
3. **Pre-treatment:** Before surface water samples are collected, rinse the bottles or samplers several times with flowing field water to minimize possible sample contamination.
4. **Sampling method:** At the chosen sampling location, the sampler is promptly immersed in water to prevent contact with other substances and to minimize the risk of airborne contamination. When sampling in water depths of 5 meters or less, samples are typically collected at a depth of 0.5 meters below the surface. For depths ranging from 5 to 10 meters, samples are collected at a depth of 0.5 meters below the surface and 0.5 meters above the bottom.
5. **Number of samples:** According to the needs of the study and the requirements of laboratory tests, water samples were collected from seven different sections of the Li River. And water samples of about 500 ml to 1 litre were collected.
6. **Marking of samples:** At the time of sample collection, each sampling bottle was marked with relevant information, such as the name of the sampling site, date, time, and so on.
7. **Sample preservation:** After sampling is completed, ensure that the samples are preserved under appropriate conditions to avoid contamination or degradation of the samples. The water samples will need to be stored at 4 degrees Celsius and sent to the laboratory for spectral analysis as soon as possible.

**Fig 1:**
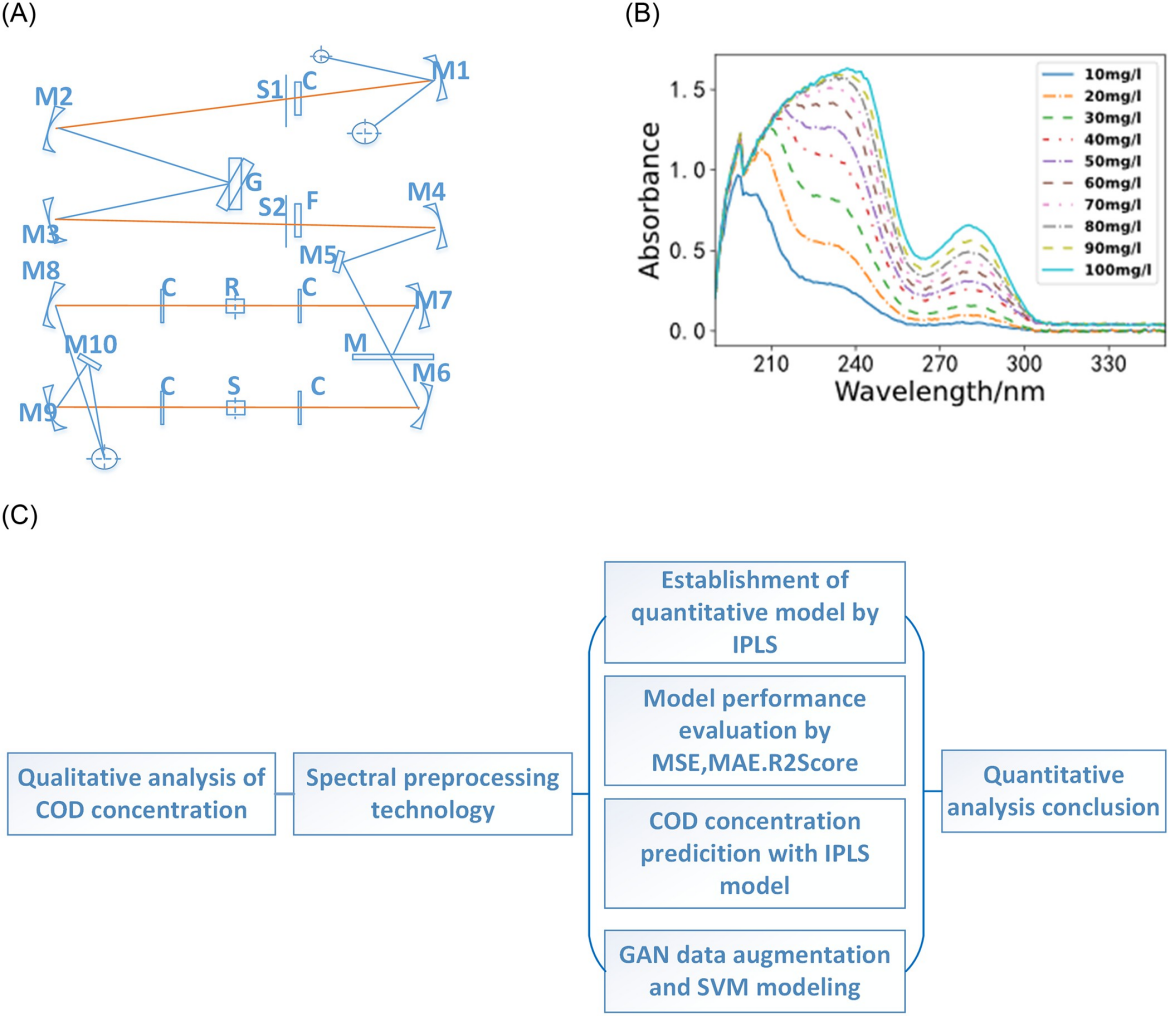
Specific implementation flowchart. (A). The internal structure of the spectrometer. (B). Spectral diagram of water sample. (C). Inversion of chemical oxygen demand model.

As shown in Fig 1, in the first step, spectra are obtained using the instrument. In the second step, model inversion research is conducted on Chemical Oxygen Demand (COD) using the Interval Partial Least Squares (IPLS), Support Vector Machine (SVM), and Generative Adversarial Networks (GAN) methods.

Fig 1A illustrates the internal structure of the spectrometer, while Fig 1B depicts the spectrum of the water sample. The spectrometer is used to collect spectral data, which is then utilized to create a spectrum for qualitative analysis of solution concentration. Fig 1C illustrates the process of qualitative analysis for COD. Initially, six methods are used for data preprocessing. Subsequently, the IPLS algorithm is used to model and predict the preprocessed data, followed by the application of GAN networks to augment data for SVM model construction. Evaluation metrics for the model include Mean Squared Error (MSE), Mean Absolute Error (MAE), and R2score.

### Spectral collection and pretreatment

**Fig 2:**
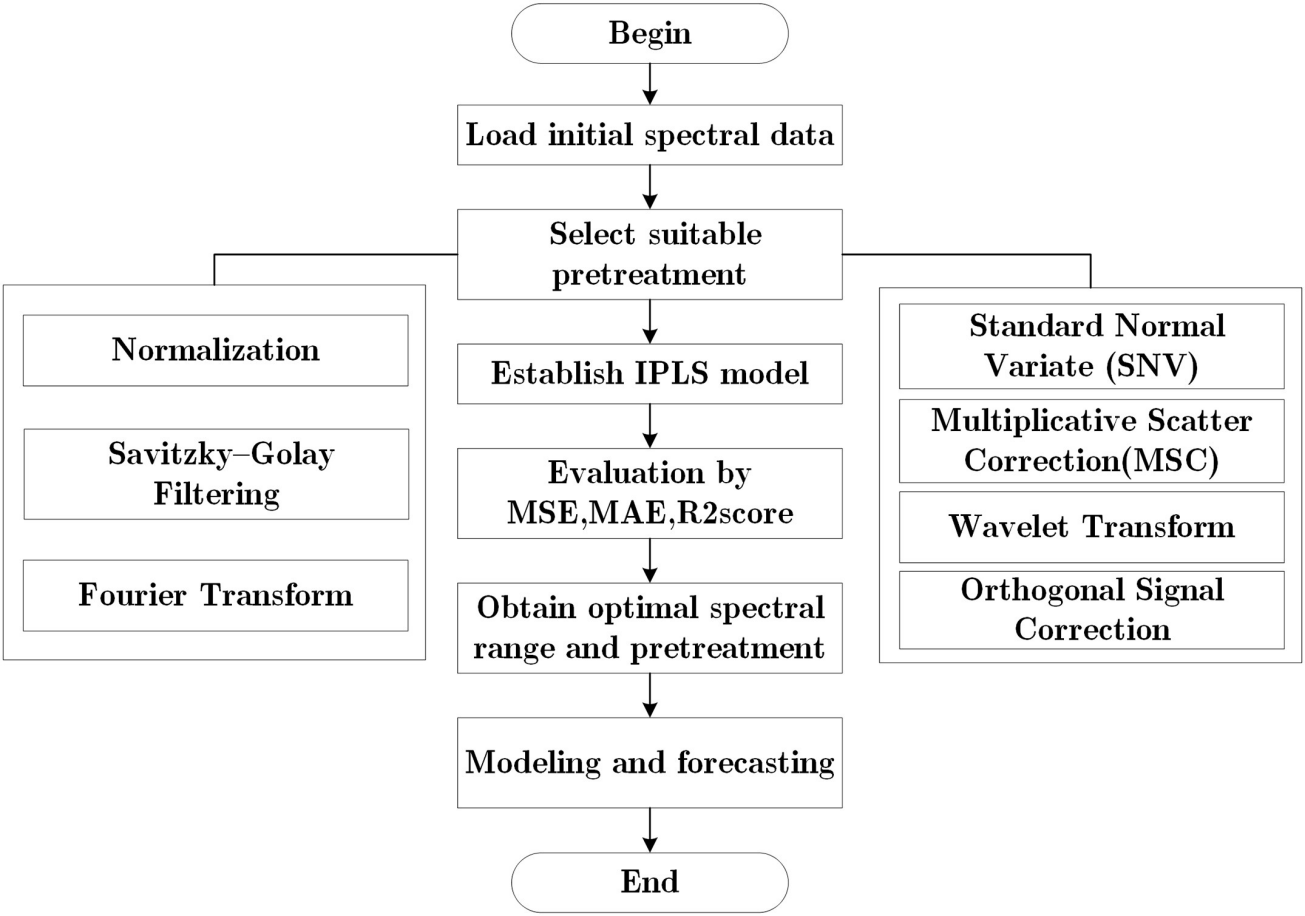
Flow chart of data pretreatment.

Spectral data collection is susceptible to noise, and therefore, pretreatment is essential. Pretreatment aids in noise elimination and reduces the impact of other factors on the model’s accuracy. As depicted in Fig 2, we have outlined common spectral preprocessing methods. This research considers six pretreatment methods, including Standard Normal Variate (SNV), Multiple Scatter Correction (MSC), Vector Normalization, Savitzky-Golay smoothing filtering (SG), Wavelet Transform (WT), and Standardization methods.

The original spectra are processed using methods to visualize them and establish an IPLS model. Based on the model’s effectiveness, the optimal pretreatment method is selected. The flowchart of pretreatment is presented in Fig 2.

## Method

### Interval partial least squares algorithm

Before introducing the IPLS algorithm, it is essential to introduce PLS, which is a typical mathematical optimization algorithm used to study the statistical relationship between the dependent variable and the independent variable. It can be employed for regression modeling when the number of sample points is less than the number of variables or severe multicollinearity among the independent variables. Previous research [39, 40] has shown that, compared to other linear models, PLS has better prediction results in qualitative analysis of UV spectra.

Before using PLS, it is essential to understand its basic principles and advantages. PLS projects the original independent variable data onto the direction of the dependent variable to obtain a new set of independent variables, thereby eliminating the multicollinearity between the independent variables. This can improve the stability and predictive ability of modeling. For example, the concentration matrix of COD can be set as the dependent variable, denoted as *Y* = (*y*_*ij*_)_*n×m*_ while the measured UV spectral absorbance matrix can set as the independent variable, represented as *X* = (*x*_*ij*_)_*n×p*_, where n is the number of water samples, m is the number of components, and p is the number of spectral wave points. We decompose X and Y into feature vectors, as shown below.


Y=UQ+G (1)

X=TP+F (2)

As shown above, U is the concentration characteristic factor matrix of n rows and d columns, Q is the *d* × *m* order concentration loading matrix; T is the UV absorbance characteristic factor matrix of n rows and d columns, P is the *d* × *p* UV absorbance loading matrix; G and F are the *n* × *m* concentration residual matrix and *n* × *p* UV absorbance residual matrix, respectively.

We decompose Y and X according to the correlation of eigenvectors to build a regression model, as shown below.


U=TB+Fd (3)

Fd is the random error matrix, and B is the d-dimensional diagonal regression coefficient matrix, for the water sample, if the measured UV absorbance vector is x, then the concentration y can be derived from the following equation.


$$y=x{\left(UX\right)}^{\prime }BQ$$ (4)

The IPLS is to build several models for spectra ranges based on the PLS, and to evaluate the models using three metrics: Mean Square Error (MSE), Mean Absolute Error (MAE) and R2score. The equations of these metrics are shown below. denotes the sample size, y denotes the true value, and $\hat{y}$ denotes the predicted value.


$$MSE(y,\hat{y})=\frac{1}{m}\sum_{i=1}^{m}(y_{i}-{\hat{y}}_{i}{)}^{2}$$ (5)

$$MAE(y,\hat{y})=\frac{1}{m}\sum_{i=1}^{m}\left|y_{i}-{\hat{y}}_{i}\right|$$ (6)

$$R^{2}=1-\frac{\sum {\left(y-\hat{y}\right)}^{2}}{\sum {\left(y-\bar{y}\right)}^{2}}$$ (7)

IPLS is chosen for its capability to effectively handle collinear spectral data. By dividing the spectral range into intervals, IPLS can capture the nonlinear relationship between spectral variables and chemical properties, thus mitigating the effects of collinearity. Moreover, IPLS allows for the selection of informative spectral intervals, focusing modeling efforts on the most relevant spectral regions. This feature enhances model interpretability and reduces computational complexity, making IPLS a suitable choice for chemical oxygen demand (COD) modeling with spectral data.

### SVM regression algorithm

The Support Vector Machine (SVM) Regression Algorithm is typically used in spectral analysis. It is an effective method to construct a nonlinear discriminant model. An introduction to SVM-based spectrum modeling is provided here.

1. Data acquisition and preprocessing: Spectral data with various compositions is collected, containing reflectance or absorption intensities at multiple wavelengths. The raw spectral data is preprocessed, including noise removal, baseline correction, and spectral smoothing etc. The preprocessing aims to enhance the quality and resolvability of the data.
2. Feature extraction: The features are extracted from the preprocessed spectral data. In spectral modeling, features are usually reflection or absorption values at various wavelengths in the spectrum.
3. Model construction: The dataset is divided into a training set and a test set. Techniques such as cross-validation are used to ensure the reliability of the model. The appropriate kernel functions for SVM, such as linear, polynomial, or Gaussian kernel, are chosen based on the specific problem. The SVM model is trained using the training set, and the model parameters are adjusted to achieve optimal results. During the training process, it will search for an optimal hyperplane that maximizes the margin between sample points.
4. Model evaluation and optimization: The trained model is evaluated using a test set, and the model performance is assessed using metrics such as accuracy, Mean Absolute Error (MAE), Mean Squared Error (MSE), and R-squared score (R2score). The model is optimized based on the evaluation results, such as adjusting hyperparameters, increasing training samples, and employing other methods.

Overall, SVM-based spectral modeling is comprehensive and involves multiple steps, including data acquisition, preprocessing, feature extraction, model construction, evaluation, and optimization. Through these steps, a spectral analysis model is constructed to address practical problems. It is chosen as the modeling algorithm due to its robustness in handling high-dimensional data with limited samples. SVM can effectively model nonlinear relationships between spectral features and COD concentrations while avoiding overfitting, even with a relatively small dataset. Additionally, SVM offers flexibility in kernel selection, allowing the modeling of complex relationships between spectral variables and target variables. This versatility makes SVM suitable for capturing the relationships present in spectral data for COD prediction.

In this manuscript, the parameters of the SVM model are shown below.

1. **’kernel’:** the default kernel function is ’rbf ’, depending on the case, we can choose ’linear’, ’sigmoid’, ’poly’ and ’precomputed’, etc. The kernel can transform a nonlinear problem into a linear one;
2. **’C’: **the penalty parameter of c-svc, whose default value is 1.0, when its value is larger, the weaker the generalization ability of the model, but when its value is lower, the stronger the generalization ability of its model;
3. **’degree’:** when setting the kernel to ’poly’, the dimensionality of ’poly’ can be set using the ’degree’ parameter, whose default value is 3;
4. **’gamma’:** the kernel coefficients for ’rbf’, ’poly’ and ’’sigmoid’, whose default value is ’auto’;
5. **’catch_size’:** the default value is 200, which denotes the size of the kernel function cache.

The selection of proper parameters in the SVM model is essential to model accuracy. Although the parameters can be set empirically, it can be time-consuming and require significant effort. Alternatively, the GridSearchCV can be employed to identify the optimal combination of hyperparameters, such as ’kernel’, ’gamma’, ’degree’, and ’C’.

The GridSearchCV performs a systematic search of the parameter space by evaluating the model’s performance. This enables the identification of the best combination that results in the higher model accuracy. To further enhance the accuracy of the model, cross-validation techniques are employed in combination with GridSearchCV.

### Generative adversarial networks

Generative adversarial networks (GAN) is one of the most classical network models in recent years and have achieved significant success in computer vision and natural language processing, etc. The main principle of GAN is to generate optimal samples in generators and discriminators using game theory.

The number of samples has an influence on the training of the model, and in this paper, due to spectral samples limitation, a GAN is used to generate samples for better training.

**Fig 3:**
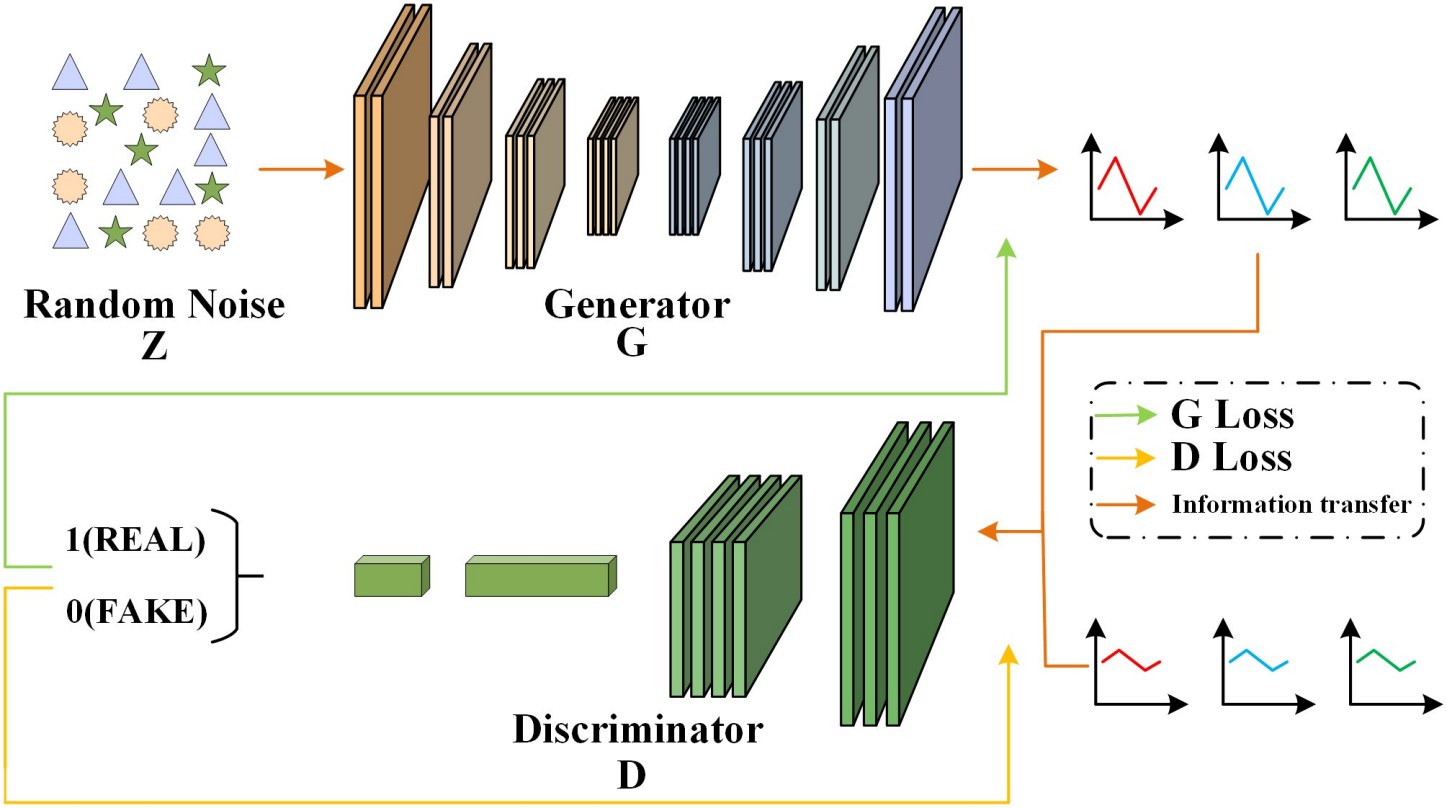
GAN network structure diagram.

As depicted in Fig 3, the generator accepts a set of random vectors and is responsible for generating realistic data, and the discriminator is responsible for learning to determine the authenticity of the data. The optimization objective function of the network is shown below.


$$\begin{array}{l} \underset{G}{\text{min}}\;\underset{D}{\text{max}}V(D,G)=E_{x\sim P_{data}(x)}[\text{log}(D(x))]+\\ E_{z\sim P_{z}(z)}[\text{log}(1-D(G(z)))] \end{array}$$ (8)

As shown above, *D* represents the Discriminator.*G* represents the Generator, where the real data *X* matches the *P*_*data*_(*x*) distribution, and *Z* represents the noisy data, which matches the *P*_*Z*_(*z*) distribution. *V*(*D*,*G*) denotes the degree of difference between the real sample and the generator sample. max_*D*_ *V*(*D*,*G*) denotes the degree of maximizing the difference between the real and generated samples when Generator is fixed, and $\underset{G}{min}\underset{D}{\;max}\;V\left(D,G\right)$ denotes the degree of minimizing the difference between the real and generated samples when Discriminator is fixed.

The training process of the generator is as follows: when the discriminator is fixed, the generator generates samples for it. At first, due to the discrepancy between the generated and real samples, the discriminator feeds the training losses to the generator. The ultimate objective is to train the generator to produce samples that are indistinguishable from real data, fooling the discriminator into classifying them as real with a high degree of confidence (i.e., close to 1).


$$\underset{G}{\text{min}}\;V(D,G)=E_{z\sim P_{z}(z)}[\text{log}(1-D(G(z)))]$$ (9)

During the training process of the discriminator, the generator is fixed and the discriminator improves its discrimination capability by continuously comparing the real samples with the generated samples. Finally, it attains a higher frequency discrimination performance. Therefore, the frequency discrimination ability of non-real samples is close to 0.


$$\begin{array}{l} \underset{D}{\text{max}}\;V(D,G)=E_{x\sim P_{data}(x)}[\text{log}(D(x))]+\\ E_{z\sim P_{z}(z)}[\text{log}(1-D(G(z)))] \end{array}$$ (10)

During the training process, both the generator and the discriminator become stronger and gradually reach a balance.

When employing a GAN network to generate one-dimensional data, both the generator and discriminator are designed as neural networks optimized for processing one-dimensional data. The following outlines the specific training process:

1. **Data preparation:** First, prepare authentic COD one-dimensional data and record the corresponding COD concentrations. The column dimension of the input data is 160, which corresponds to the total number of spectral data sampling points.
2. **Initialize the network:** Randomly initialize the weights and biases of the generator and discriminator.
3. **Define the loss function:** For generating one-dimensional data, use ’binary_crossentropy’ as the loss function for both the discriminator and generator. Additionally, utilize ’rmsprop’ (Root Mean Square Prop) as the optimizer for both networks. The generator’s loss function aims to ensure that the generated data distribution closely matches the distribution of real data, while the discriminator’s loss function aims to correctly distinguish between real and generated data.
4. **Train the Discriminator:** In each training iteration, sample a batch of data from the original real data and generate a batch of fake data using the Generator. Merge two batches and assign labels (1 for real data and 0 for generated data). Then, feed the merged samples into the Discriminator, calculate its loss, and update parameters through backpropagation.
5. **Train the Generator:** Generate a batch of fake data from the generator and feed it into the discriminator. The objective here is to have the generated samples misclassified as real data (labeled 1) by the discriminator. Calculate the generator’s loss and update its parameters through backpropagation, improving the generator’s ability to produce realistic samples.
6. **Adversarial training:** During the training process, the generator and discriminator confront each other. The generator attempts to produce realistic COD samples to deceive the discriminator, while the discriminator strives to distinguish real data from the generated data. This adversarial training process continues for 500 iterations.
7. **End of training:** The training process concludes when a certain number of iterations are reached or when the performance of the generator and discriminator stabilizes.
8. **Data generation:** After training is complete, the generator can be employed to generate new one-dimensional data. By sampling from the generator, one can obtain one-dimensional data samples that match the generated model.

In this paper, GAN network consists of Generators and Discriminators, first 3 kinds of Generator network parameters are introduced as shown in Table 2 below.

**Table 2:** Three generator network parameters.

Layer	Type			
	Input Shape	Output Shape	Number of Parameters	Activation
Input	(None, 160)	(None, 160)	0	None
Dense1	(None, 160)	***(None*, *5/10/20)***	***805/1610/3220***	ReLU
Dense2	**(None, 5/10/20)**	**(None, 5/10/20)**	**30/110/420**	ReLU
Dense3	**(None, 5/10/20)**	**(None, 160)**	**960/1760/3360**	tanh

As shown in the Table 2, all three Generators consist of four layers: Input, Dense1, Dense2, and Dense3. Their network structure is quite similar. To facilitate display, their network parameters are presented in a table. Taking the Output Shape of Dense1 layer as an example, the Output Shape of the Dense1 layer for three Generators is depicted by the values (None, 5/10/20), which corresponds to (None, 5), (None, 10), and (None, 20), respectively. These values indicate that the output dimensions after processing by the Dense1 layer of the three Generators are (None, 5), (None, 10), and (None, 20). Likewise, ‘805/1610/3220’ denotes the number of network parameters in the Dense1 layer for the three Generators, which are 805, 1610, and 3220, respectively.

As shown in the Table 3, all three Discriminators consist of four layers: Input, Dense1, Dense2, Dropout and Dense3. Their network structure is also quite similar. Taking the Input Shape of Dense3 layer as an example, the Input Shape of the Dense3 layer for three Discriminator is depicted by the values (None, 5/10/20), which corresponds to (None, 5), (None, 10), and (None, 20), respectively. These values indicate that the input dimensions after processing by the Dense3 layer of the three Discriminators are (None, 5), (None, 10), and (None, 20). Likewise, ‘805/1610/3220’ denotes the number of network parameters in the Dense3 layer for the three Discriminator, which are 6, 11, and 21, respectively.

**Table 3:** Three discriminator network parameters.

Layer	Type			
	Input Shape	Output Shape	Number of Parameters	Activation
Input	(None, 160)	(None, 160)	0	None
Dense1	(None, 160)	**(None, 5/10/20)**	**805/1610/3220**	ReLU
Dense2	**(None, 5/10/20)**	**(None, 5/10/20)**	**30/110/420**	ReLU
Dropout	**(None, 5/10/20)**	**(None, 5/10/20)**	0	None
Dense3	***(None*, *5/10/20)***	(None, 1)	***6/11/21***	Sigmoid

In summary, the Generator model takes a 160-dimensional vector as input, processes it through two hidden layers with several units each and ReLU activation, and then produces a 160-dimensional output vector with each element being the result of applying the hyperbolic tangent (tanh) function. The discriminator model takes a 160-dimensional vector as input and processes it through two hidden layers with several units each and ReLU activation functions. It then applies dropout to the outputs of the second layer, followed by a final dense layer with a sigmoid activation function to produce a single output representing the probability of the input being classified as the positive class in a classification task.

GAN are selected for data augmentation to overcome the challenge of limited datasets. It can produce synthetic spectral data that mimic the distribution of real spectral samples. This process expands the training dataset and enhances model generalization. Unlike traditional data augmentation methods such as interpolation or oversampling, it can generate diverse and realistic spectral variations, capturing the complexity and variability of real-world spectral data more effectively.

### GridSearchCV technique

GridSearchCV is a commonly used technique for parameter tuning in machine learning. It combines cross-validation and grid search to efficiently search for optimal parameters. By specifying a range of parameters to explore, it systematically evaluates the performance of different parameter combinations using cross-validation.

The process begins with the initialization of hyperparameter combinations. Subsequently, an SVM model is established, and the various parameter combinations are sequentially traversed and evaluated for their modeling effectiveness. Each parameter combination is inputted into the SVM model, and the modeling process is completed. Finally, the best-performing parameter combination, which yields the most favorable modeling results, is selected.

In this paper, we utilize GridSearchCV to fine-tune the parameters of the SVM model. By exhaustively searching through all possible combinations within the specified parameter range, we aim to identify the optimal parameter configuration that maximizes the model’s performance in cross-validation. This approach ensures that the chosen parameter values are well-suited to the problem at hand, enhancing the overall effectiveness and reliability of the SVM model.

## Results and discussion

### Spectral pretreatment

**Fig 4:**
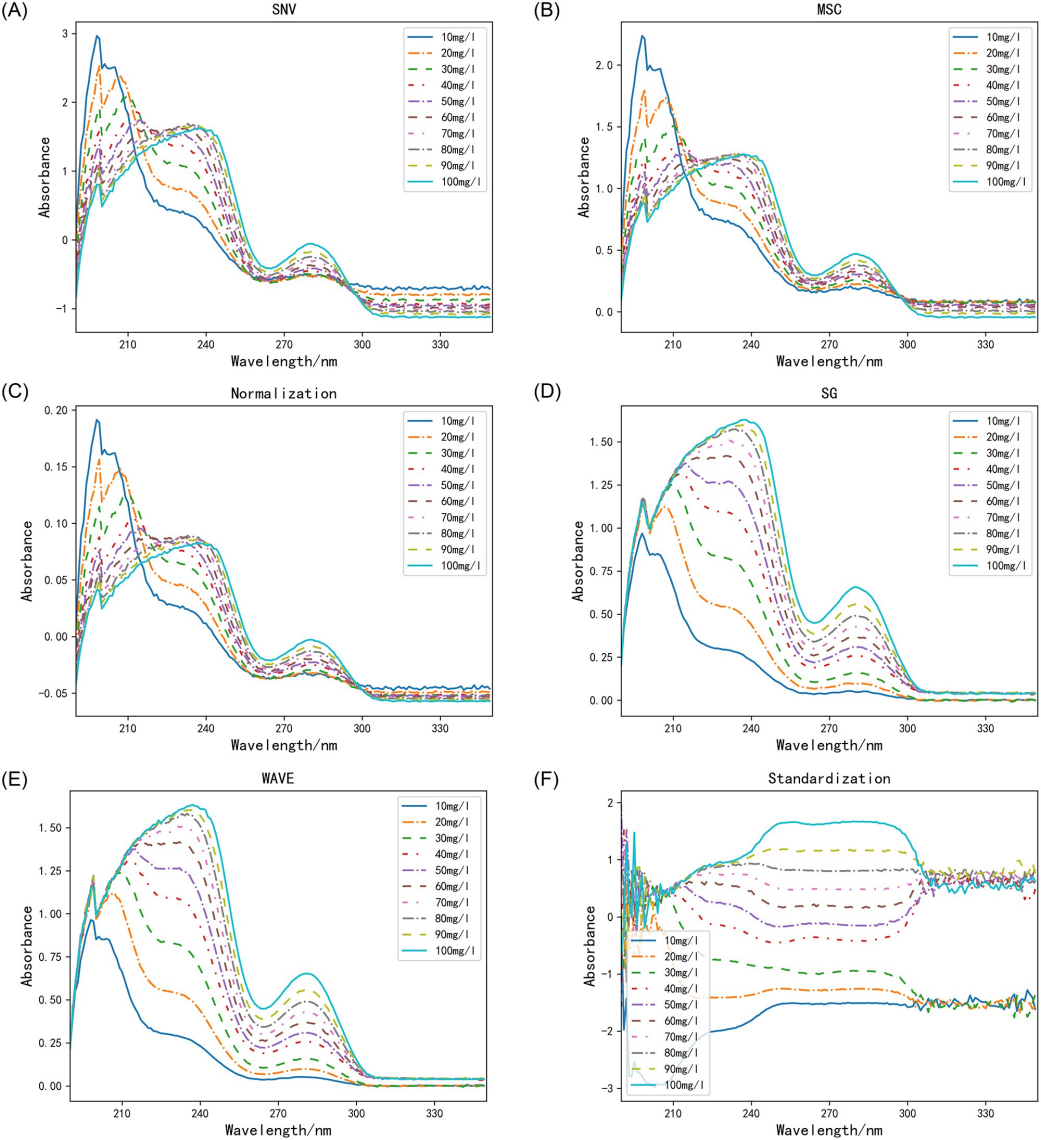
Spectra obtained after pretreatment.

Spectral pretreatment can remove irrelevant information such as noise, and it is useful to analyze the correlation between the spectrum and the COD concentration. The results of pretreatment using various methods are shown in Fig 4 below.

As illustrated in the Fig 4, the effectiveness of pretreatment by using Standard Normal Variate (SNV), Multiple Scattering Correction (MSC), Normalization, Savitzky-Golay Smoothing Filtering (SG), Wavelet Transformation (WAVE) and Standardization methods for the original spectra, as shown in the Fig 4. The spectral graph obtained by SNV, MSC, and normalization methods are relatively similar, while the spectral graph obtained by SG and WAVE methods also exhibit similarities. The spectral range from 190 to 340 nm is plotted on the horizontal axis, while the absorbance values are shown on the vertical axis. The application of pretreatment enhances the smoothness of the original spectra.

### Feature wavelength selection

#### Interval partial least squares method

After acquiring the pre-processed data, the selection of the wavelength range is executed using the IPLS method. To determine the optimal spectral range, the 190 nm~350 nm spectral range is divided into ten subintervals of equal width, and a PLS is performed on each subinterval, thereby establishing individual regression models. Subsequently, the model exhibiting superior performance is selected. *log*_10_ (*MSE*), *log*_10_ (*MAE*) and R2score are used as metrics of the model.

**Fig 5:**
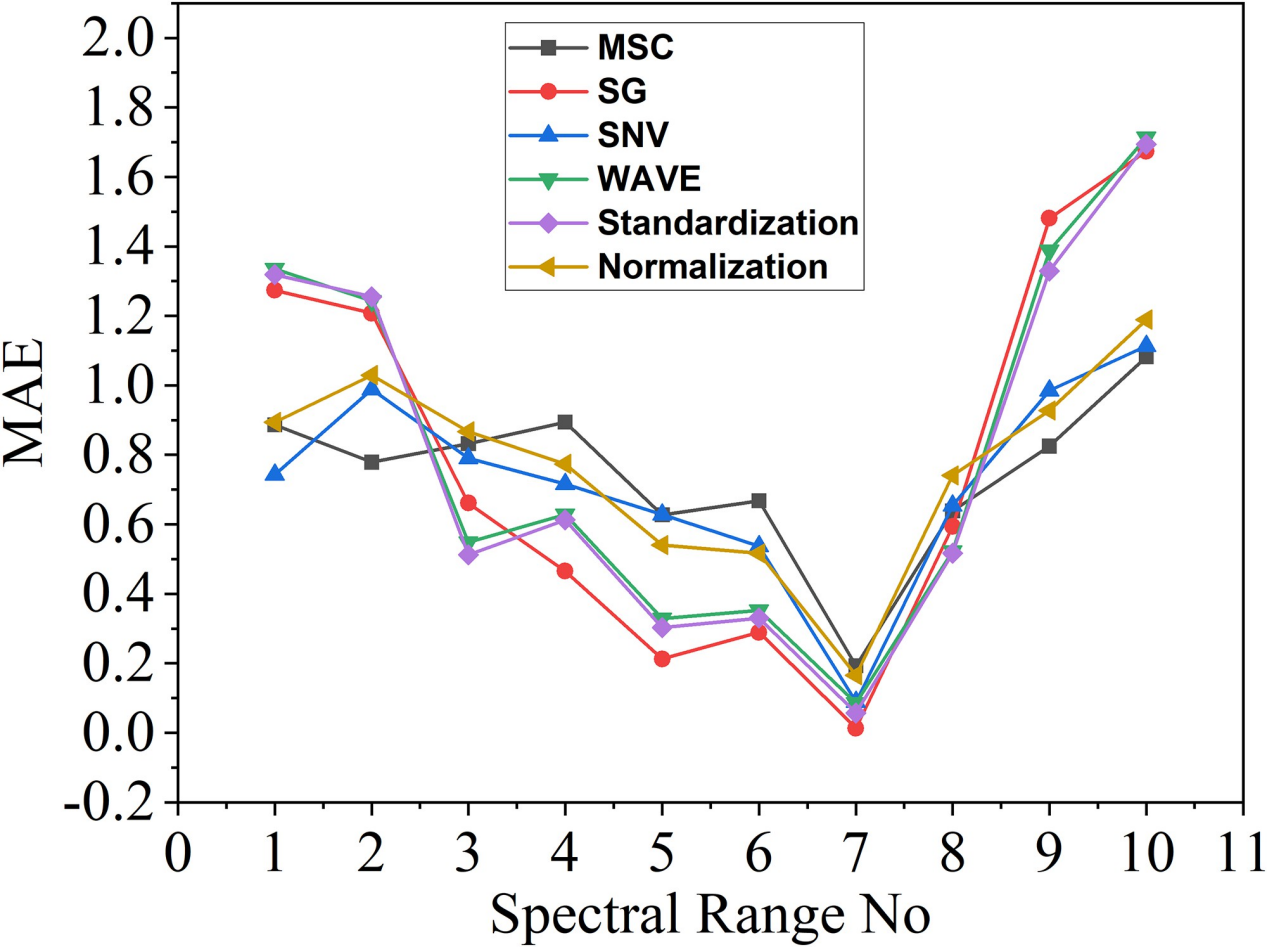
Graph of MAE value results.

As shown in Table 4 above, the MAE obtained in different pretreatment methods and modeling in different spectral ranges are given. It shows that all six methods achieve the minimum MAE value in the seventh range of the spectrum, while the values on both sides increase sequentially. As indicated in the "Original modeling effect" column of the table, the model achieved the best modeling effect in the seventh range without data preprocessing, with the *log*_10_ *MAE* of 0.0278, highlighted in bold in the table. From the value of *log*_10_ *MAE*, the final MAE of the original model can be found to be 1.0661. Specifically, taking the value of 0.0136 in the 7th row and 3rd column as an example, the original spectrum was initially pre-processed using the SG method, which yielded the input for the PLS in the 7th spectral range. This value represents the *log*_10_ *MAE* of the model. The final MAE of the model processing by the SG method is 1.0318. Better results can be attained by modeling the data with the pretreatment method. The data are also visually depicted in the Fig 5.

**Table 4:** Table of MAE corresponding to different pretreatment methods.

Spectral Range No	Original modeling effect	Pretreatment methods					
		MSC	SG	SNV	WAVE	Standardization	Normalization
1	1.2112	0.8864	1.2736	0.7429	1.3348	1.3184	0.8940
2	1.1954	0.7790	1.2077	0.9887	1.2429	1.2552	1.0291
3	0.5725	0.8322	0.6614	0.7901	0.5479	0.5124	0.8671
4	0.4766	0.8945	0.4654	0.7163	0.6281	0.6127	0.7736
5	0.2665	0.6275	0.2126	0.6280	0.3284	0.3027	0.5401
6	0.3735	0.6683	0.2890	0.5371	0.3526	0.3305	0.5166
7	**0.0278**	0.1927	**0.0136**	0.0886	0.0861	0.0573	0.1655
8	0.5210	0.6385	0.5937	0.6541	0.5225	0.5174	0.7407
9	1.5046	0.8251	1.4809	0.9847	1.3880	1.3296	0.9277
10	1.7103	1.0825	1.6740	1.1133	1.7135	1.6940	1.1895

As illustrated in the Fig 5, the horizontal axis displays each spectral range, numbered 1 to 10, and the vertical axis shows the MAE in each range. The smallest error value is obtained for the 7th range, specifically for the wavelength range of 238–253 nm. Regarding the pretreatment methods, the SG method yielded better results than the other methods.

The MSE resulting from modeling using different pretreatment methods and spectral ranges are presented in the Table 5 below. The values in the table represent *log*_10_ *MSE*.

**Table 5:** Table of MSE corresponding to different pretreatment methods.

Spectral Range No	Original modeling effect	Pretreatment methods					
		MSC	SG	SNV	WAVE	Standardization	Normalization
1	2.5581	1.9370	2.7123	1.6644	2.7776	2.7489	2.0513
2	2.5510	1.7487	2.5502	2.1463	2.6000	2.6302	2.3167
3	1.2502	1.8283	1.4709	1.8236	1.2302	1.1650	1.9401
4	1.1494	1.9766	1.1413	1.6248	1.4578	1.4300	1.7308
5	0.7105	1.4695	0.5993	1.4500	0.8677	0.8136	1.2896
6	0.9320	1.5336	0.8051	1.2588	0.9200	0.8870	1.2108
7	**0.2172**	0.5235	**0.1681**	0.3172	0.3096	0.2512	0.4467
8	1.2145	1.4689	1.3510	1.5038	1.2014	1.1963	1.6714
9	3.2844	1.8916	3.2123	2.2463	3.0375	2.9037	2.0964
10	3.6246	2.4304	3.5384	2.4235	3.6445	3.6013	2.5904

As shown above, the MSE obtained in different pretreatment methods and modeling in different spectral ranges are given. Table 5 presents the MSE corresponding to the model established using six preprocessing methods. It shows that all six methods achieve the minimum MSE value in the seventh range, while the values on both sides increase sequentially. As indicated in the "Original modeling effect" column of the Table 5, the model achieved the best modeling effect in the seventh range without data preprocessing, with the *log*_10_ *MSE* of 0.2172, highlighted in bold in the Table 5. From the value of *log*_10_ *MSE*, the final MSE of the original model can be found to be 1.6489. Specifically, taking the value of 0.1681 in the 7th row and the 3rd column as an example, the original spectrum was initially pre-processed using the SG method, which yielded the input for the PLS in the 7th spectral range. This value represents the MSE of the model.

**Fig 6:**
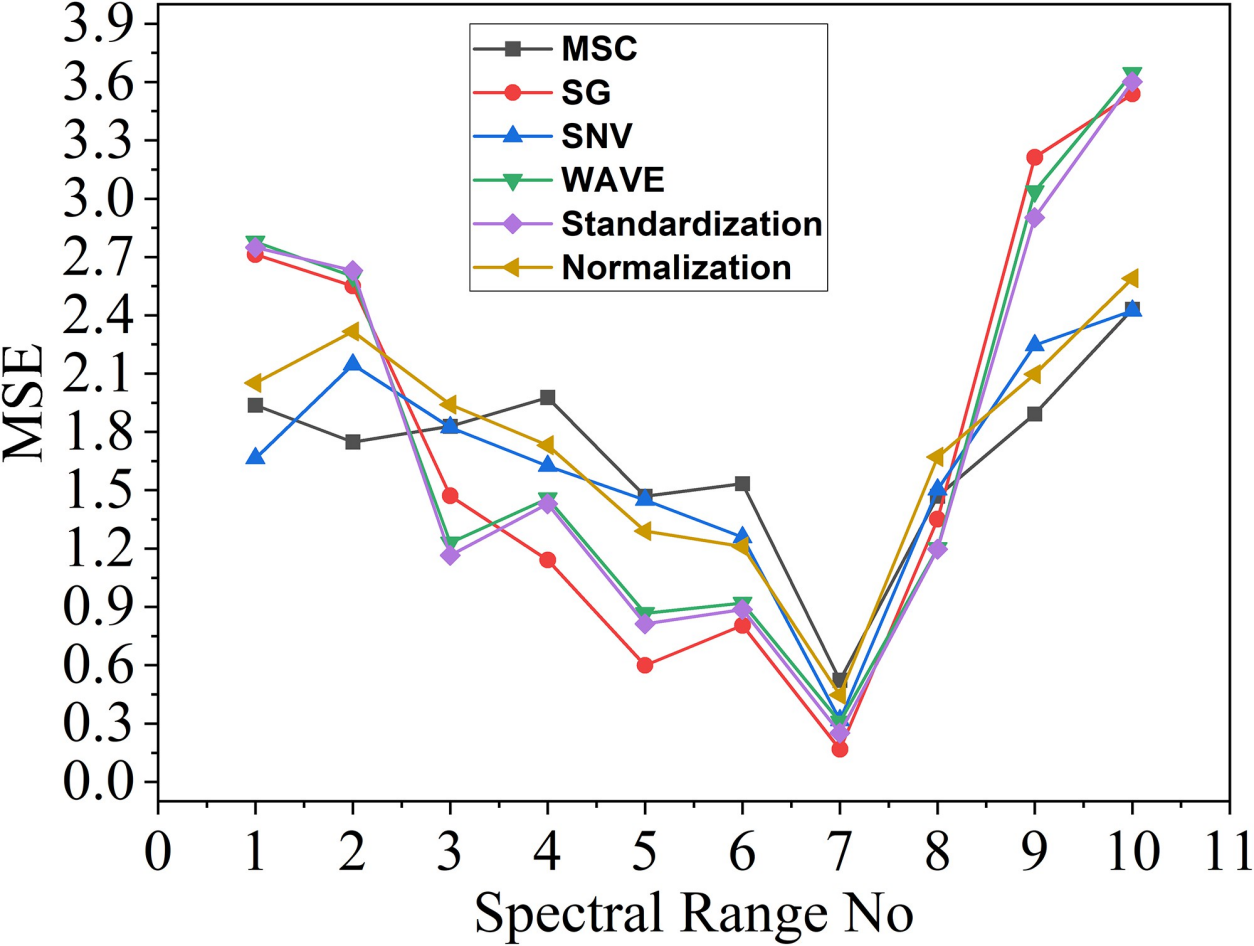
Graph of MSE value results.

Our analysis indicates that there are significant variations in the errors of models constructed across different spectral intervals. Specifically, the 10th subinterval generates the largest model error, while the 7th subinterval results in the smallest model error. Additionally, better results can be attained by modeling the data using the pretreatment method. In the 7th range, from the smallest value of *log*_10_ *MSE*, the final MSE of the original data can be found to be 1.6489 and the MSE of the model constructed by the SG method is 1.4727. The data are also visually depicted in the Fig 6.

Fig 6 displays the results of the MSE. Overall, the MSE values are slightly larger than the MAE, and the overall tendency of the MSE is similar to the MAE. The data trends in the two graphs are similar, with only numerical differences. The six preprocessing methods all achieve the minimum error value in the seventh range of the spectrum, while the values on both sides increase sequentially. The MSE obtained through the 7th range modeling is the smallest, and the SG method demonstrates a superior pretreatment effect compared to other methods.

The data of R2score are shown below. The "-" in the Table 6 indicates some instances with poor modeling results.

**Table 6:** Table of R2score corresponding to different pretreatment methods.

Spectral Range No	Original modeling effect	Pretreatment methods					
		MSC	SG	SNV	WAVE	Standardization	Normalization
1	0.2596	0.7390	-	0.8215	-	-	0.6238
2	0.9066	0.8426	-	0.6441	-	-	0.4467
3	0.9628	0.8106	0.1167	0.7808	0.1833	0.2509	0.6477
4	0.9888	0.5527	0.8837	0.4997	0.6066	0.6951	0.7354
5	0.9811	0.9284	0.9902	0.9108	0.9743	0.9782	0.9448
6	0.9940	0.9040	0.9829	0.9513	0.9697	0.9741	0.9428
7	0.9620	0.9740	**0.9942**	0.9940	0.9876	0.9896	0.9925
8	-	0.7456	0.8813	0.7622	0.9239	0.9290	0.3752
9	**-**	0.1187	-	-	-	-	-
10	0.2596	-	-	0.0563	-	-	-

The results presented in the Table 6 demonstrate that six pretreatment methods yield improved performance in the seventh range, as evidenced by R2score values, while the values on both sides increase and decrease sequentially. The correlation modeled in the 7th range is the strongest, while the correlation modeled in other ranges deteriorates. In comparison, the model constructed for the 7th subinterval has the optimal effect. Specifically, the SG method achieves the highest R2score value of 0.9942 for modeling in the seventh range, while the original modeling effect closely follows with an R2score value of 0.9620.

**Fig 7:**
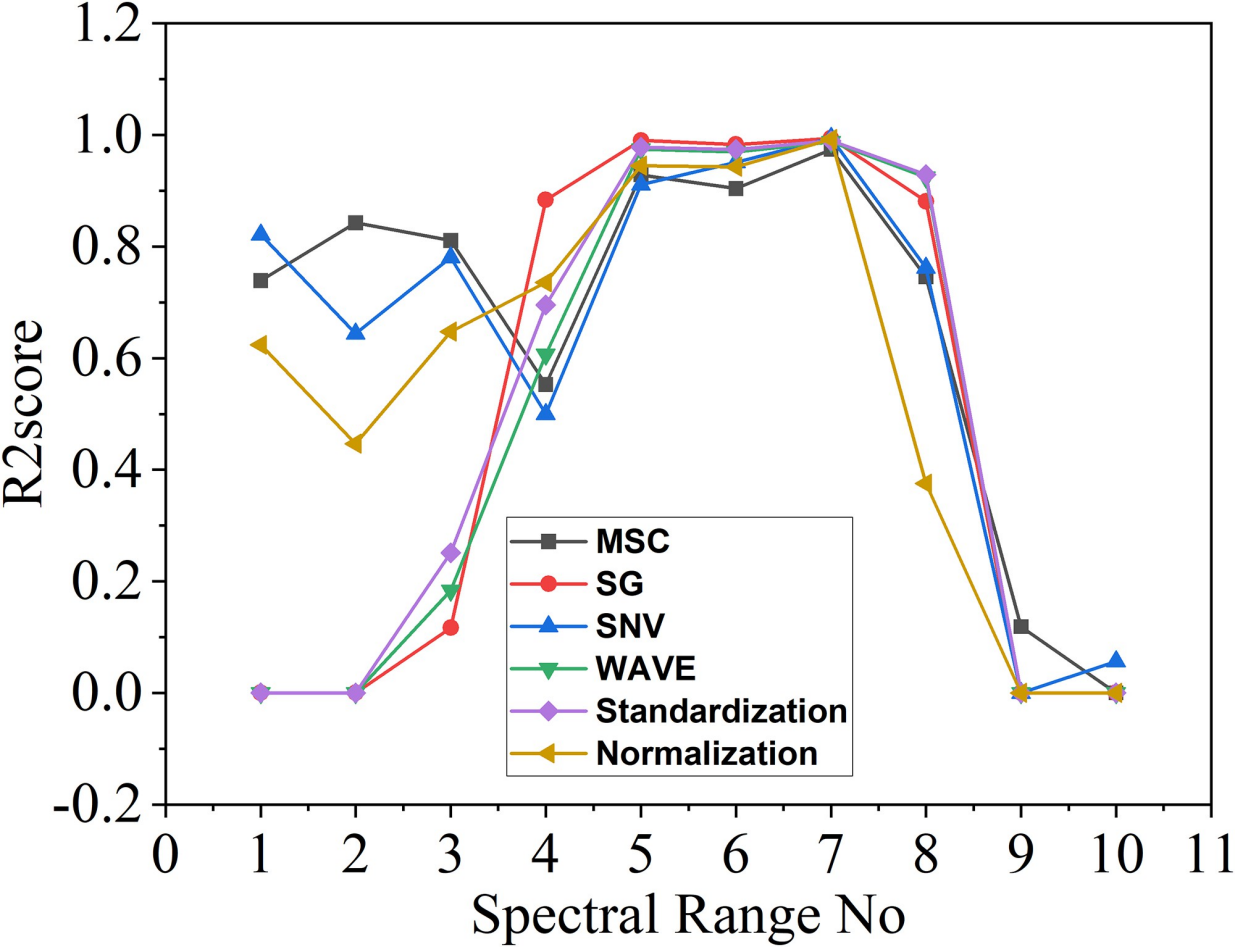
Graph of R2score value results.

As illustrated in the Fig 7, the vertical axis represents the R2score values obtained from the different pretreatment methods across various spectral ranges, where a value closer to 1 indicates better performance. Among the pretreatment methods, the SG method outperformed the others in the seventh spectral range.

Finally, we evaluate three indexes to determine the optimal pretreatment method for samples. The results indicate that the SG pretreatment method is most effective. Furthermore, we determined that the optimal modeling range was the 7th segment, which to a range of 238~253 nm. A PLS model is established based on the spectral data, which could be used for the subsequent inversion study.

### Spectral inversion study

**Fig 8:**
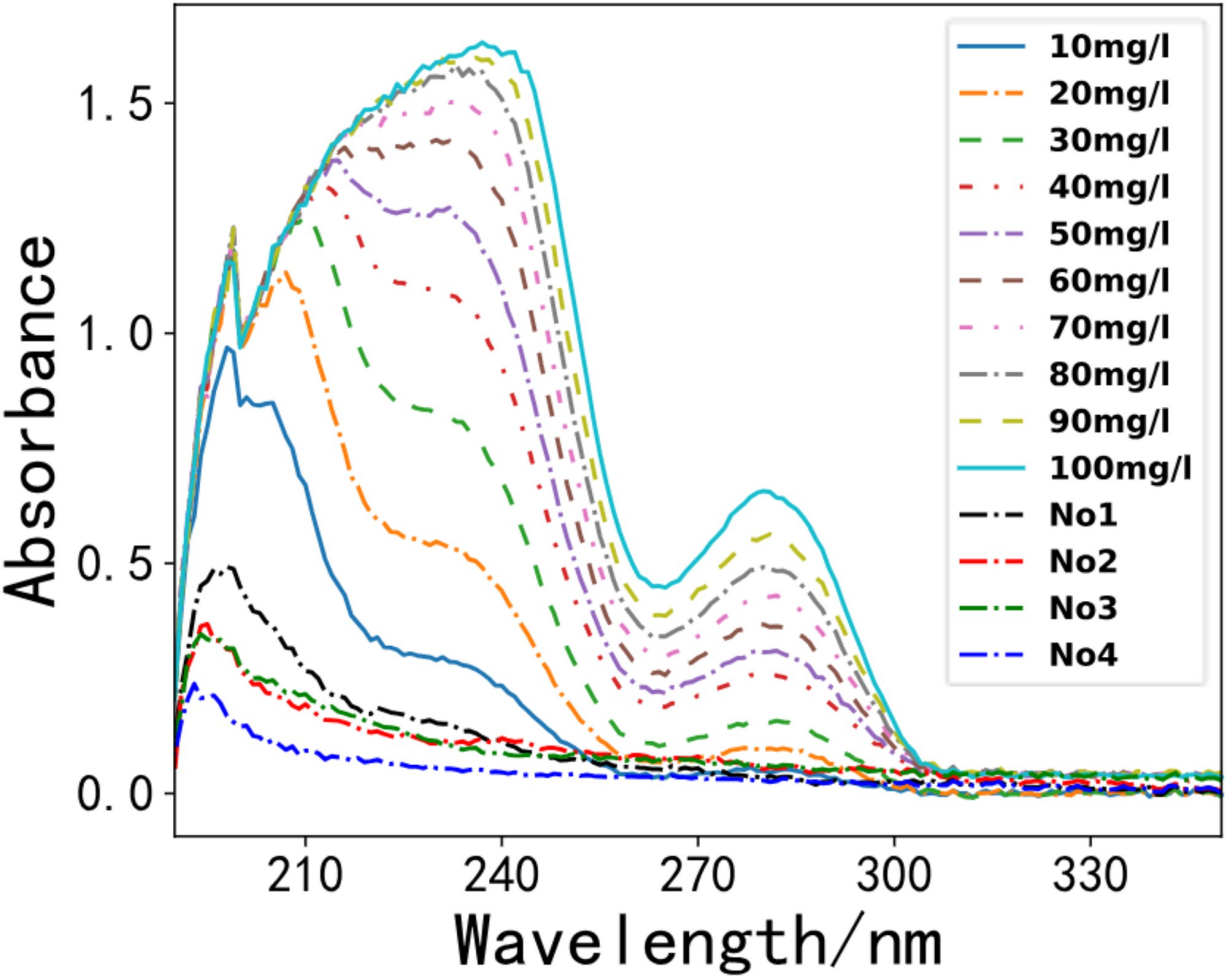
Comparison of the first 4 water samples with the standard solution.

**Fig 9:**
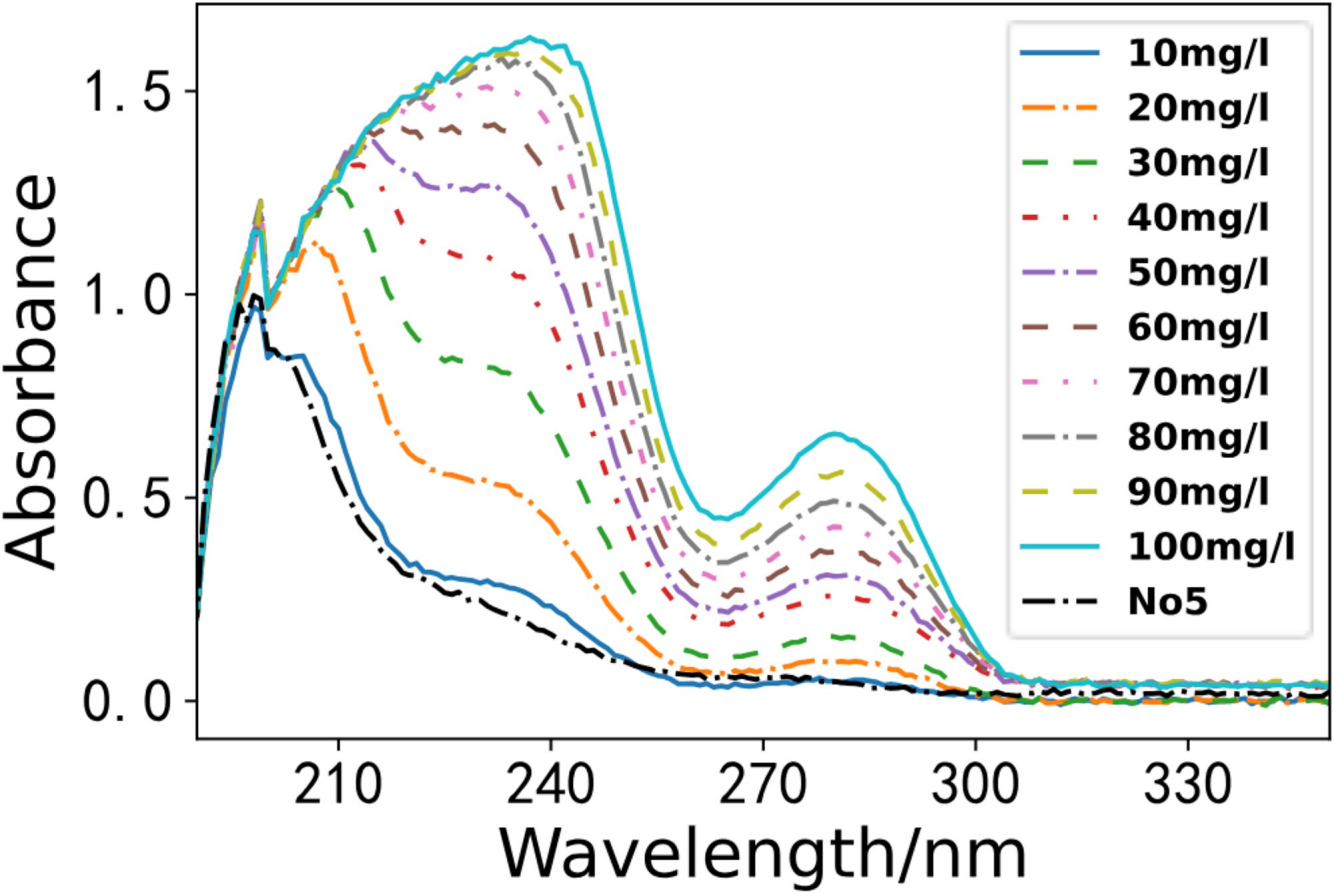
Comparison of water sample 5 and standard solution.

**Fig 10:**
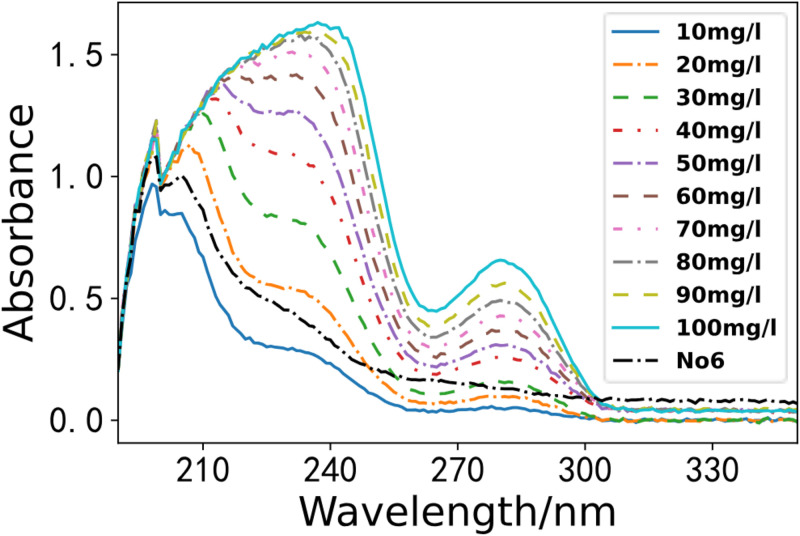
Comparison of water sample 6 and standard solution.

**Fig 11:**
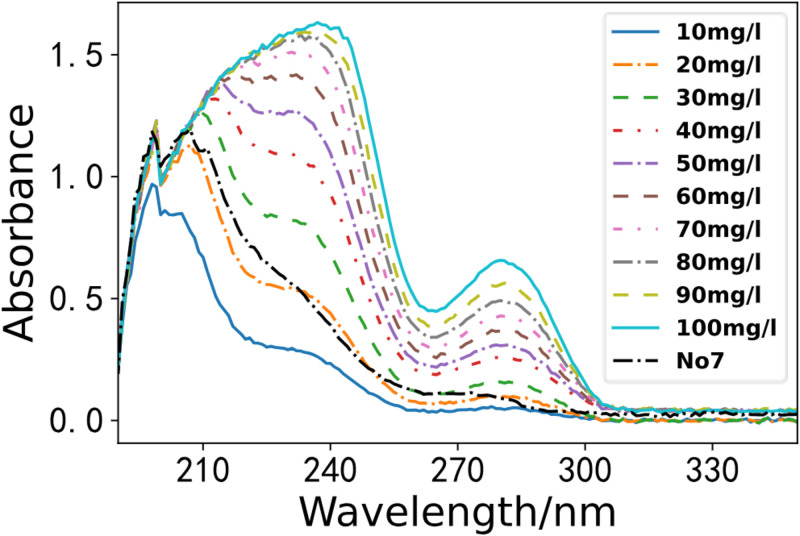
Comparison of water sample 7 and standard solution.

According to the water sample collection criteria in Environmental Monitoring, we gathered seven water samples from a section of the Li River. We utilized the optimal PLS model for the spectral inversion study. As depicted in Figs 8–11. These four figures depict the spectral lines of various water samples and COD standard solutions. By comparing the spectral lines, the concentration of the water sample can be preliminarily determined.

In the first step of our analysis, we compared the absorbance curves of the water samples with the standard COD solutions, as illustrated in Fig 8. The spectrogram presents the absorbance values of the four water samples as indicated by the black, red, green, and blue dashed lines. Notably, the spectral lines of the four water samples show a closer resemblance.

As illustrated in the Fig 8, these four water samples have no obvious absorption peaks in the overall spectral range, and the preliminary analysis shows that the concentration of COD is low in these water samples.

Water sample 5 is analyzed, and the results are presented in Fig 9. The spectral line depicts as the black dashed line, exhibits a close resemblance to the standard solution of COD with a concentration of 10 mg/L.

As illustrated in the Fig 10, we compared the water sample 6 with standard solutions. The absorbance curve is depicted by the black dashed line in Fig 10. Analysis of the spectrum indicates that the COD of water sample 6 falls between the spectra of the COD standard solutions of 10 mg/L and 20 mg/L, with a concentration of approximately 15 mg/L.

As illustrated in the Fig 11, we compared water sample 7 with standard solutions. Our analysis shows that the COD in water sample 7 is greater than that in water sample 6. The absorbance curve is depicted by the black dashed line in Fig 11. Notably, the spectrum of water sample 7 is more like the spectrum of the COD standard solution with a concentration of approximately 20 mg/L.

As illustrated in the Fig 11, we used the PLS model to predict the COD concentrations of the water samples. The results infer that the COD concentrations of the first four water samples were lower, with concentrations of 10.87 mg/L for water sample 5, 14.88 mg/L for water sample 6, and 19.29 mg/L for water sample 7. Notably, the model predictions were consistent with the qualitative analysis results.

This consistency offers ways to confirm the model’s accuracy. The reliability of modeling is improved by consistency in a number of ways, including the following:

1. Check the model’s correctness: Since spectral data figures are analyzed for qualitative analysis, model outputs that align with these findings suggest that the model has a higher degree of accuracy. We can confirm whether the model accurately represents the components in the water sample by contrasting the expected results with the findings of the qualitative investigation.
2. Optimize the features chosen and the model’s parameters: Consistency analysis can be used to evaluate how well the features and parameters chosen for the model worked. In the event when the model’s output is consistent with qualitative analysis, the selected features and parameters might be more appropriate. On the other hand, if there are variations, feature selection or model parameter selection might need to be reconsidered.
3. Increasing the model’s credibility in real-world applications: Reliable outcomes strengthen the model’s argument and increase its credibility in real-world applications. In contexts like water sample prediction, the model’s credibility plays a critical role in enabling decision-making and appropriate action.

Consistency with qualitative analysis results can be viewed as an indicator of model quality and reliability. This approach can be adopted when high precision in the model is not a strict requirement.

### SVM-based cod modeling

The original and pre-processed spectral data are fed into the GAN network to generate synthetic data for modeling purposes. Next, we use the SVM model to train on and evaluate against the original data, the generated data, and a mix of both. Through this process, we can verify the feasibility of the GAN in generating synthetic data for modeling.

The original data, the data obtained by pretreatment and the data generated by GAN are used as data sets for the training of SVM, and the modeling process is shown below. The initial parameter array of the SVM is set in Table 7. Table 7 lists various kernel functions and their respective parameters. Each function includes a ’C’ parameter, which represents the degree parameter of c-svc. The default value is 1.0. A higher value enhances the generalization ability of the model, while a lower value strengthens the model’s generalization ability. The gamma parameter signifies the kernel coefficients for ’rbf’, ’poly’, and ’sigmoid’, with the default value being ’auto’.

**Table 7:** Table of initial parameter arrays.

Kernel	Parameter array
’linear’	’C’: [2, 3, 5, 7, 8, 9, 10, 11, 13]
’rbf ’	’C’: [1, 2, 3, 5, 7, 8, 9, 10, 11] ’gamma’: [1e-10, 1e-8, 1e-7, 1e-6, 1e-5, 1e-4, 1e-3, 1e-2, 1e-1, 2e-1,3e-1,4e-1,5e-1,6e-1, 1, 1/n_features]
’poly’	’C’: [3, 10, 15, 18] ’gamma’: [1e-4, 1e-3, 1e-2, 1e-1,2e-1,3e-1,4e-1, 1, 1/n_features] ’degree’: [5, 7, 9, 11]
’sigmoid’	’C’: [1, 1.5, 2, 3, 5, 7, 8, 9, 10, 11, 13, 15, 50, 100] ’gamma’: [1e-8, 1e-7, 1e-6, 1e-5, 1e-4, 1e-3, 1e-2, 1e-1, 1, 1/n_features]

As previously mentioned, in our SVM modeling approach, we utilize four different kernel functions: ’rbf’, ’sigmoid’, ’poly’, and ’linear’. The ’gamma’ parameter, which is only valid for ’rbf’, ’poly’, and ’sigmoid’, has a default value of ’1/n_features’, where ’n_features’ is the number of sample features. Additionally, the ’degree’ parameter has a default value of 3. The values of the evaluation indexes without searching for parameters are shown in Table 8.

**Table 8:** Table of evaluation index values.

Evaluation index	Type			
	linear	rbf	sigmoid	poly
Accuracy	0.056	0.055	0.001	0.001
MSE	300.000	500.000	711.111	611.111
MAE	14.443	18.888	23.333	21.111
R2score	0.594	0.318	0.023	0.165

Table 8 displays the effectiveness of utilizing four kernel functions for SVM modeling. Based on the evaluation of the model performance using four indicators, it shows that the MSE and MAE of the model are larger, while the accuracy and correlation of the model are lower. As observed from the results, the performance of our SVM is suboptimal, likely due to the limited size of spectral datasets and the lack of parameter tuning. To address this issue, we apply the GridSearchCV method to perform a thorough parameter search. We initialize the search with the parameter array shown in Table 9 and utilize the GridSearchCV to systematically probe the parameters and identify the optimal combination of them.

**Table 9:** Table of the optimal parameters obtained from GridSearchCV.

Optimal parameters	Type			
	linear	rbf	sigmoid	poly
C	8	7	50	3
gamma	-	0.3	0.01	0.1
degree	-	-	-	5

As indicated in Table 9 of the manuscript, the GridSearchCV yielded the optimal parameters. Automatic parameter adjustment with GridSearchCV yields optimal results for a given set of parameters. Specifically, each kernel function’s parameter lists—such as the "C" and "gamma" lists—are supplied. In order to discover the best combination of parameters, GridSearchCV searches the parameter list exhaustively and trains the SVM model for each combination. Subsequently, the ideal parameters are obtained and employed to train the SVM model, leading to enhanced training results.

After obtaining the optimal parameters, they are passed into the SVM for modeling, and the results are shown in Table 10.

**Table 10:** Effects of modeling using parameters from the GridSearchCV.

Optimal parameters	Type			
	linear	rbf	sigmoid	poly
Accuracy	**0.555**	0.333	0.222	0.389
MSE	**183.333**	472.222	972.222	822.222
MAE	**8.333**	13.889	21.666	16.667
R2score	**0.747**	0.349	-0.340	-0.134

The results in Table 10 demonstrate a significant improvement in the model’s performance after the parameter search, as compared to the results presented in Table 8. After adjusting the parameters, the linear kernel performs relatively the best in the modeling process using four kernel functions. For instance, the ’sigmoid’ kernel’s accuracy is observed to be lower, with a corresponding ’C’ value of 50. It is noteworthy that a higher ’C’ value tends to influence the model’s generalization. Taking linear kernels as an example, the correlation and accuracy have increased, while the MSE and MAE values have decreased.

A key element of SVM is the kernel function. The kernel function enables SVM to perform nonlinear mapping in high-dimensional space, thereby resolving the issue of linear inseparability in the original feature space. Four kernel functions are utilized for modeling in this article. An examination of the modeling effect is presented below.

Firstly, non-linearly separable data in the original feature space can be handled with the "rbf" function. In order to adapt to various complex data distributions, it enables the learning of increasingly complex decision boundaries.

Second, the "sigmoid" kernel function is sensitive to parameter choice and appropriate in scenarios with extremely complex data distributions. Moreover, data exhibiting polynomial relationships in the feature space is well-suited for "poly" kernel function. They can adapt to various data distributions by adjusting the offset and order of the polynomials. The "linear" kernel can perform better when the data relationship is relatively simple and does not require intricate nonlinear mapping. The spectral data in this manuscript demonstrate a small-scale, simple data distribution and no complex data distribution in the feature space. As a result, the "linear" kernel function is utilized to enhance modeling results.

Overall, the comprehensive evaluation criteria indicate that the ’linear’ kernel outperforms the other alternatives, making it the preferred choice for subsequent modeling.

After selecting the ’linear’ kernel, the data are generated utilizing three GAN networks with different structures, and the GAN-generated data are aggregated with the original data for training, and the results are shown in Table 11.

**Table 11:** Effects of modeling using parameters from the GridSearchCV.

Evaluation index	Type			
	Original	GAN1	GAN2	GAN3
Accuracy	0.555	0.571	0.619	0.619
MSE	183.333	109.524	114.286	104.761
MAE	8.333	6.190	5.714	5.714
R2score	0.747	0.882	0.877	0.887

As shown in Table 11, the original spectral data and the generated data were blended for SVM modeling using 3 types of GAN for data augmentation, and then carrying out SVM training, compared with modeling directly with the original data, the MSE and MAE of the model have considerably decreased, and the accuracy and R2score of the model have considerably increased, the accuracy has improved by 2.88%, 11.53% and 11.53% in turn, and the R2score has improved by 18.07%, 17.40% and 18.74% in turn.

Due to the small dataset, the samples used for training is relatively limited, only approaching a hundred. SVM did not exhibit good performance on this small dataset. That’s why using GAN for data augmentation. After data augmentation, the modeling effect is better than before. To achieve better results, more data needs to be generated. In conclusion, GAN provides a better way for data augmentation when the model is trained with less data and upgrades the training effect to a certain extent.

## Conclusion

Spectral collinearity and limited spectral data set are two main problems affecting COD modeling. To address these problems, First, the IPLS method is utilized to effectively identifies the spectral range for modeling and mitigates the impact of spectral collinearity. Secord, we used six different data preparation techniques, the model fits best in the 7th range (238~253 nm), according to the results. From modeling without data pretreatment, the MSE, MAE, and R2score values are 1.6489, 1.0661, and 0.9942, respectively. Following the SG method’s pretreatment, the R2score rises to 0.9944 and the MSE and MAE drop to 1.4727 and 1.0318, respectively. This suggests that proper data pretreatment is essential to obtain more reliable results in spectroscopic analysis.

Next, we predicted the concentrations in the water sample using the best model. The findings show that the first four water samples had lower COD values, but samples 5, 6, and 7 had amounts of 10.87 mg/L, 14.88 mg/L, and 19.29 mg/L, respectively. Notably, the results of the qualitative study align with the model predictions.

To address the problem of having a little dataset, we used three different GANs for data augmentation before using the results for SVM modeling. The experimental results indicated that the MSE and MAE of SVM models decreased when compared to the original dataset. Additionally, the R2score grew by 18.07%, 17.40%, and 18.74%, and the accuracy of the three models improved by 2.88%, 11.53%, and 11.53%.

In summary, IPLS, GAN, and SVM are selected based on their complementary strengths in addressing the challenges of spectral collinearity and limited datasets. IPLS addresses collinearity by emphasizing informative spectral intervals, GAN enhances the dataset with realistic synthetic samples, and SVM efficiently models relationships between spectral features and COD concentrations.

This research still has several limitations despite making some progress. The model can be applied to some simple water systems. And the sub-models described in the manuscript can serve as the basis for future research. Some explicit directions and hypotheses for future studies are as follows.

1. **Investigating different methods of data augmentation:** We can explore alternative approaches to data augmentation, such as variational autoencoders, autoencoders, or different versions of generative adversarial networks. And we can compare the performance of various strategies on small-sample datasets.
2. **Expanding modeling to other indicators of water quality:** We can utilize the suggested model to model indicators of other water quality, such as total phosphorus, ammonia nitrogen, etc. This extension would confirm the applicability and versatility of the approach.
3. **Examining multimodal data fusion:** To enhance the modeling accuracy of water quality indicators in the future, we can consider integrating multimodal data, such as sensor and spectral data. It would be beneficial to incorporate multiple types of data into a single model and evaluate how they impact the model’s performance.
